# The interaction of vision and audition in two-dimensional space

**DOI:** 10.3389/fnins.2015.00311

**Published:** 2015-09-17

**Authors:** Martine Godfroy-Cooper, Patrick M. B. Sandor, Joel D. Miller, Robert B. Welch

**Affiliations:** ^1^Advanced Controls and Displays Group, Human Systems Integration Division, NASA Ames Research CenterMoffett Field, CA, USA; ^2^San Jose State University Research FoundationSan José, CA, USA; ^3^Institut de Recherche Biomédicale des Armées, Département Action et Cognition en Situation OpérationnelleBrétigny-sur-Orge, France; ^4^Aix Marseille Université, Centre National de la Recherche Scientifique, ISM UMR 7287Marseille, France

**Keywords:** visual-auditory, localization, precision, accuracy, 2D, MLE

## Abstract

Using a mouse-driven visual pointer, 10 participants made repeated open-loop egocentric localizations of memorized visual, auditory, and combined visual-auditory targets projected randomly across the two-dimensional frontal field (2D). The results are reported in terms of variable error, constant error and local distortion. The results confirmed that auditory and visual maps of the egocentric space differ in their precision (variable error) and accuracy (constant error), both from one another and as a function of eccentricity and direction within a given modality. These differences were used, in turn, to make predictions about the precision and accuracy within which spatially and temporally congruent bimodal visual-auditory targets are localized. Overall, the improvement in precision for bimodal relative to the best unimodal target revealed the presence of optimal integration well-predicted by the Maximum Likelihood Estimation (MLE) model. Conversely, the hypothesis that accuracy in localizing the bimodal visual-auditory targets would represent a compromise between auditory and visual performance in favor of the most precise modality was rejected. Instead, the bimodal accuracy was found to be equivalent to or to exceed that of the best unimodal condition. Finally, we described how the different types of errors could be used to identify properties of the internal representations and coordinate transformations within the central nervous system (CNS). The results provide some insight into the structure of the underlying sensorimotor processes employed by the brain and confirm the usefulness of capitalizing on naturally occurring differences between vision and audition to better understand their interaction and their contribution to multimodal perception.

## Introduction

The primary goal of this research was to determine if and to what extent the precision (degree of reproducibility or repeatability between measurements) and accuracy (closeness of a measurement to its true physical value) with which auditory (A) and visual (V) targets are egocentrically localized in the 2D frontal field predict precision and accuracy in localizing physically and temporally congruent, visual-auditory (VA) targets. We used the Bayesian framework (MLE, Bülthoff and Yuille, 1996; Bernardo and Smith, 2000) to test the hypothesis of a weighted integration of A and V cues (1) that are not equally reliable and (2) where reliability varies as a function of direction and eccentricity in the 2D frontal field. However, this approach does not address the issue of the differences in reference frames for vision and audition and the sensorimotor transformations. We show that analyzing the orientation of the response distributions and the direction of the error vectors can provide some clues to solve this problem. We first describe the structural and functional differences between the A and V systems and how the CNS realizes the merging of the different spatial coordinates. We then review evidence from psychophysics and neurophysiology that sensory inputs from different modalities can influence one another, suggesting that there is a *translation mechanism* between the spatial representations of different sensory systems. We then reviewed the Bayesian framework for multisensory integration, which provides a set of rules to optimally combine sensory inputs with variable reliability. Finally, we present a combined quantitative and qualitative approach to test the effect of *spatial determinants* on integration of spatially and temporally congruent A and V stimuli.

### Structural and functional differences between the visual and the auditory systems

The inherent structural and functional differences between vision and audition have important implications for bimodal VA localization performance. First, A and V signals are represented in different neural encoding formats at the level of the cochlea and the retina, respectively. Whereas vision is tuned to spatial processing supported by a 2D retinotopic (eye-centered) spatial organization, audition is primarily tuned to frequency analysis resulting in a tonotopic map, i.e., an orderly map of frequencies along the length of the cochlea (Culler et al., 1943). As a consequence, the auditory system must derive the location of a sound on the basis of acoustic cues that arise from the geometry of the head and the ears (binaural and monaural cues, Yost, 2000).

The localization of an auditory stimulus in the horizontal dimension (azimuth, defined by the angle between the source and the forward vector) results from the detection of left-right interaural differences in time (interaural time differences, ITDs, or interaural phase differences, IPDs) and differences in the received intensity (interaural level differences, ILDs, Middlebrooks and Green, 1991). To localize a sound in the vertical dimension (elevation, defined by the angle between the source and the horizontal plane) and to resolve front-back confusions, the auditory system relies on the detailed geometry of the pinnae, causing acoustic waves to diffract and undergo direction-dependent reflections (Blauert, 1997; Hofman and Van Opstal, 2003). The two different modes of indirect coding of the position of a sound source in space (as compared to the direct spatial coding of visual stimuli) result in differences in spatial resolution in these two directions. Carlile (Carlile et al., 1997) studied localization *accuracy* for sound sources on the sagittal median plane (SMP), defined as the vertical plane passing through the midline, ±20° about the auditory-visual horizon. Using a head pointing technique, he reported constant errors (*CE*s) as small as 2-3° for the horizontal component and between 4 and 9° for the vertical component (see also Oldfield and Parker, 1984; Makous and Middlebrooks, 1990; Hofman and Van Opstal, 1998; for similar results). For frontal sound sources (0° position in both the horizontal and vertical plane), Makous and Middlebrooks reported *CE*s of 1.5° in the horizontal plane and 2.5° in the vertical plane. The smallest errors appear to occur for locations associated with the audio-visual horizon, also referred to as horizontal median plane (HMP) while locations off the audio-visual horizon were shifted toward the audio-visual horizon, resulting in a compression of the auditory space that is exacerbated for the highest and lowest elevations (Carlile et al., 1997). Such a bias has not been reported for locations in azimuth. Recently, Pedersen and Jorgensen (2005) reported that the size of the *CE*s in the SMP depends on the actual sound source elevation and is about +3° at the horizontal plane, 0° at about 23° elevation, and becomes negative at higher elevations (e.g., −3° at about 46°; see also Best et al., 2009).

For *precision*, variable errors (*VE*s) are estimated to be approximately 2° in the frontal horizontal plane near 0° (directly in front of the listener) and 4–8° in elevation (Bronkhorst, 1995; Pedersen and Jorgensen, 2005). The magnitude of the *VE* was shown to increase with sound source laterality (eccentricity in azimuth) to a value of 10° or more for sounds presented on the sides or the rear of the listener, although to a lesser degree than the size of the *CE*s (Perrott et al., 1987). For elevation the *VEs* are minimum at frontal location (0°, 0°) and maximum at the extreme positive and negative elevations.

On the other hand, visual resolution, contrast sensitivity, and perception of spatial form fall off rapidly with eccentricity. This effect is due to the decrease of the density of the photoreceptors in the retina (organized in a circular symmetric fashion) as a function of the distance from the fovea (Westheimer, 1972; DeValois and DeValois, 1988; Saarinen et al., 1989). Indeed, humans can only see in detail within the central visual field, where spatial resolution (acuity) is remarkable (Westheimer, 1979: 0.5°; Recanzone, 2009: up to 1 to 2° with a head pointing task). The visual spatial resolution varies also consistently at isoeccentric locations in the visual field. At a fixed eccentricity, *precision* was reported to be higher along the HMP (where the cones density is highest) than along the vertical (or sagittal) median plane (vertical-horizontal anisotropy, VHA). Visual localization was also reported to be more precise along the lower vertical meridian than in the upper vertical meridian (vertical meridian asymmetry, VMA) a phenomenon that was also attributed to an higher cone density in the superior portion of the retina which processes the lower visual field (Curcio et al., 1987) up to 30° of polar angle (Abrams et al., 2012). These asymmetries have also been reported at the level of the lateral geniculate nucleus (LGN) and in the visual cortex. It is interesting to note that visual sensitivity at 45° is similar in the four quadrants and intermediate between the vertical and the horizontal meridians (Fuller and Carrasco, 2009). For *accuracy*, it is well-documented that a brief visual stimulus flashed just before a saccade is mislocalized, and systematically displaced toward the saccadic landing point (Honda, 1991). This results in a symmetrical compression of visual space (Ross et al., 1997) known as “foveal bias” (Mateeff and Gourevich, 1983; Müsseler et al., 1999; Kerzel, 2002) and that has been attributed to an oculomotor signal that transiently influences visual processing (Richard et al., 2011). Visual space compression was also observed in perceptual judgment tasks, where memory delays were involved, revealing that the systematic target mislocalization closer to the center of gaze was independent of eye movements, therefore demonstrating that the effect was perceptual rather than sensorimotor (Seth and Shimojo, 2001).

These fundamental differences in encoding are preserved as information is processed and passed on from the receptors to the primary visual and auditory cortices, which raises a certain number of issues for visual-auditory integration. First, the spatial coordinates of the different sensory events need to be merged and maintained within a common reference frame. For vision, the initial transformation can be described by a logarithmic mapping function that illustrates the correspondence between the Cartesian retinal coordinates and the polar superior colliculus (SC) coordinates. The resulting collicular map can be conceived as an eye-centered map of saccade vectors in polar coordinates where saccades amplitude and direction are represented roughly along orthogonal dimensions (Robinson, 1972; Jay and Sparks, 1984; Van Opstal and Van Gisbergen, 1989; Freedman and Sparks, 1997; Klier et al., 2001).

Conversely, for audition, information about acoustic targets in the SC is combined with eye and head position information to encode targets in a spatial or body-centered frame of reference (motor coordinates, Goossens and Van Opstal, 1999). More precisely, the representation of auditory space in the SC involves a hybrid reference frame immediately after the sound onset, that evolves to become predominantly eye-centered, and more similar to the visual representation by the time of a saccade to that sound (Lee and Groh, 2012). Kopco (Kopco et al., 2009) proposed that the coordinate frame in which vision calibrates auditory spatial representation might be a mixture between eye-centered and craniocentric, suggesting that perhaps, both representation get transformed in a way that is more consistent with the motor commands of the response to stimulations in either modality. Such a transformation would potentially facilitate VA interactions by resolving the initial discrepancy between the A and V reference frames. When reach movements are required, which involve coordinating gaze shifts with arm or hand movements, the proprioceptive cues in limb or joint reference frames are also translated into an eye-centered reference frame (Crawford et al., 2004; Gardner et al., 2008).

### Strategies for investigating intersensory interactions and previous related research

Multisensory integration refers to the processes by which information arriving from one sensory modality interacts and sometimes biases the processing in another modality, including how these sensory inputs are combined to yield to a unified percept. There is an evolutionary basis to the capacity to merge and integrate the different senses. Integrating information carried by multiple sensors provides substantial advantages to an organism in terms of survival, such as detection, discrimination, and speed responsiveness. Empirical studies have determined a set of rules (determinants) and sites in the brain that govern multisensory integration (Stein and Meredith, 1993). Indeed, multisensory integration is supported by the heteromodal (associative) nature of the brain. Multisensory integration starts at the cellular level with the presence of multisensory neurons all the way from subcortical structures such as the SC and inferior colliculus (IC) to cortical areas.

Synchronicity and spatial correspondence are the key determinants for multisensory integration to happen. Indeed, when two or more sensory stimuli occur at the same time and place, they lead to the perception of a unique event, detected, identified and eventually responded to, faster than either input alone. This multisensory facilitation is reinforced by a semantic congruence between the two inputs, and susceptible to be modulated by attentional factors, instructions or inter-individual differences. In contrast, slight temporal and/or spatial discrepancy between two sensory cues, can be significantly less effective in eliciting responses than isolated unimodal stimuli.

The manipulation of one or more parameters on which the integration of two modality-specific stimuli are likely to be combined is the privileged approach for the study of multisensory interactions. One major axis of research in the domain of multisensory integration has been the experimental conflict situation in which an observer receives incongruent data from two different sensory modalities, and still perceives the unity of the event. Such experimental paradigms, in which observers are exposed to temporally congruent, but spatially discrepant A and V targets, reveal substantial intersensory interactions. The most basic example is “perceptual fusion” in which, despite separation by as much as 10° (typically in azimuth), the two targets are perceived to be in the same place (Alais and Burr, 2004; Bertelson and Radeau, 1981; Godfroy et al., 2003). Determining exactly where that perceived location is requires that observers be provided with a response measure, for example, open-loop reaching, by which the V, A, and VA targets can be *egocentrically* localized. Experiments of this sort have consistently showed that localization of the spatially discrepant VA target is strongly biased toward the V target. This phenomenon is referred to as “ventriloquism” because it is the basis of the ventriloquist's ability to make his or her voice seem to emanate from the mouth of the hand-held puppet (Thurlow and Jack, 1973; Bertelson, 1999). It is important to note, however, that despite its typically inferior status in the presence of VA spatial conflict, audition can contribute to VA localization accuracy in the form of a small shift of the perceived location of the V stimulus toward the A stimulus (Welch and Warren, 1980; Easton, 1983; Radeau and Bertelson, 1987; Hairston et al., 2003b).

The most widely accepted explanation of ventriloquism is the *Modality Precision* or *Modality Appropriateness* hypothesis, according to which the more precise of two sensory modalities will bias the less precise modality more than the reverse (Rock and Victor, 1964; Welch and Warren, 1980; Welch, 1999). Thus it is that vision, typically more precise than audition (Fisher, 1968) and based on a more spatially articulated neuroanatomy (Hubel, 1988), is weighted more heavily in the perceived location of VA targets. This model also successfully explains “visual capture” (Hay et al., 1965) in which the felt position of the hand viewed through a light-displacing prism is strongly shifted in the direction of its visual locus. Further support for the visual capture theory was provided in an experiment by Easton (1983), who showed that when participants were directed to move the head from side to side, thereby increasing their auditory localizability in this dimension, ventriloquism declined.

Bayesian models have shown to be powerful methods to account for the optimal combination of multiple sources of information. The Bayesian model makes specific predictions, among which VA localization *precision* will exceed that of the more precise modality (typically vision) according to the formula:

(1) $$\sigma_{VA}^{2}=\frac{\sigma_{V}^{2}\sigma_{A}^{2}}{\sigma_{V}^{2}+\sigma_{A}^{2}}\leq min\left(\sigma_{V}^{2},\sigma_{A}^{2}\right)$$

where $\sigma_{A}^{2},\sigma_{V}^{2},$ and $\sigma_{VA}^{2}$, are respectively the variances in the auditory, visual, and bimodal distributions. From the variance of each modality, one may derive, in turn, their *relative weight*s, which are the normalized reciprocal variance of the unimodal distributions (Oruç et al., 2003), with respect to the bimodal percept according to the formula:

(2) $$W_{V}=\frac{\frac{1}{\sigma_{V}^{2}}}{\frac{1}{\sigma_{V}^{2}}+\frac{1}{\sigma_{A}^{2}}}and\;W_{A}=1-W_{V}$$

where *W*_*V*_ represent the visual weight and *W*_*A*_ the auditory weight. With certain mathematical assumptions, an optimal model of sensory integration has been derived based on maximum-likelihood estimation (MLE) theory. In this framework, the optimal estimation model is a formalization of the modality precision hypothesis and makes mathematically explicit the relation between the reliability of a source and it's effect on the sensory interpretation of another source. According to the MLE model of multisensory integration, a sensory source is reliable if the distribution of inferences based on that source has a relatively small variance (Yuille and Bülthoff, 1996; Ernst and Banks, 2002; Battaglia et al., 2003; Alais and Burr, 2004). In the opposite case scenario, a sensory source is regarded as unreliable if the distribution of the inferences has a large variance (noisy signal). If the noise associated with each individual sensory estimate is independent and the prior normally distributed (all stimulus positions are equally likely), the maximum-likelihood estimate for a bimodal stimulus is a simple weighted average of the unimodal estimates where the weights are the normalized reciprocal variance of the unimodal distributions:

(3) $$\hat{r_{VA}}=\left(\hat{r_{V}}W_{V}\right)+\left(\hat{r_{A}}W_{A}\right)$$

where $\hat{r_{VA}}$, $\hat{r_{V}},$ and $\hat{r_{A}}$, are respectively, the bimodal, visual and auditory location estimates and *W*_*V*_ and *W*_*A*_ are the weights of the visual and auditory stimuli.

This relation allows quantitative predictions to be made, for example, on the spatial distribution of adaptation to VA displacements. Within this framework, visual capture is simply a case in which the visual signal shows less variability in error and is assigned a weight of one as compared to the less reliable cue (audition), which is assigned a weight of zero. For spatially and temporally coincident A and V stimuli, and assuming that the variance of the bimodal distribution is smaller than that of either modality alone (Witten and Knudsen, 2005), then multisensory localization trials perceived as unified should be less variable and as accurate as localization made in the best unimodal condition. It is of interest to note that Ernst and Bülthoff (2004) considered that the term *Modality Precision* or *Modality Appropriateness* is misleading because it is not the modality itself or the stimulus that dominates. Rather, because the dominance is determined by the estimate and how reliably it can be derived within a specific modality from a given stimulus, the term “Estimate Precision” would probably be more appropriate.

Different strategies for testing intersensory interactions can be distinguished: (a) impose a spatial discrepancy between the two modalities (Bertelson and Radeau, 1981), (b) use spatially congruent stimuli but reduce the precision of the visual modality by degrading it (Battaglia et al., 2003; Hairston et al., 2003a; Alais and Burr, 2004), (c) impose a temporal discrepancy between the two modalities (Colonius et al., 2009), and (d) capitalize on inherent differences in localization precision between the modalities (Warren et al., 1983). In the present research, we used the last of these approaches by examining VA localization precision and accuracy as a function of the eccentricity and direction of physically and spatially congruent V and A targets. The effect of spatial determinants (such as eccentricity and direction) of VA integration has already been investigated, although infrequently and with many restrictions. For eccentricity, Hairston (Hairston et al., 2003b) showed that (1) increasing distance from the midline was associated with more variability in localizing temporally and spatially congruent VA targets, but not in localizing A targets and (2) that the variability in localizing spatially coincident multisensory targets was inversely correlated with the average bias obtained with spatially discrepant A and V stimuli. They didn't report a reduction in localization variability in the bimodal condition. A possible explanation for the lack of multisensory improvement in this study is that the task was limited to targets locations in azimuth, and hence, also to responses in azimuth, reducing the uncertainty of the position to one dimension. Experiments on VA spatial integration have almost always been limited to location in azimuth, with the implicit assumption that their results apply equally across the entire 2D field. Very few studies have investigated VA interactions in 2D (azimuth and elevation cues). An early experiment by Thurlow and Jack (1973) compared VA fusion in azimuth vs. in elevation, taking advantage of the inherent differences in auditory precision between these two directions. Consistent with the MLE, fusion was greater in elevation, where auditory localization precision is relatively poor, than it was in the azimuth (results confirmed and extended by Godfroy et al., 2003). Investigating saccadic eye movements to VA targets, studies also demonstrated a role of direction for VA interactions (Heuermann and Colonius, 2001).

### The present research

Beside a greater ecological valence, a 2D experimental paradigm provides the opportunity to investigate the effect of spatial determinants on multisensory integration. The present research compared the effect of direction and eccentricity on the localization of spatially congruent visual-auditory stimuli. Instead of experimentally manipulating the resolution of the A and V stimuli, we capitalized on the previously described variations in localization precision and accuracy as a function of spatial location. The participants were presented with V, A, and physically congruent VA targets in each of an array of 35 spatial locations in the 2D frontal field and were to indicate their perceived egocentric location by means of a mouse-controlled pointer in an open-loop condition (i.e., without any direct feedback of sensory-motor input–output). Of interest were the effects of spatial direction (azimuth and elevation) and eccentricity on localization *precision* and *accuracy* and how these effects may predict localization performance for the VA targets. Following Heffner's conventions (Heffner and Heffner, 2005), we distinguished between localization precision, known as the statistical (variable) error (*VE*) and the localization bias (sometimes called localization accuracy), or the systematic (constant) error (*CE*). The specific predictions of the experiment were:

#### Precision (VE)

Based on the MLE model, localization precision for the VA targets will exceed that of the more precise modality, which by varying amounts across the 2D frontal field is expected to be vision. Specifically, the contribution of the visual modality to bimodal precision should be greater toward the center of the visual field than in the periphery. Response variability was also used to provide insight about the performance of the sensory motor chain. Indeed, a greater level of variability in the estimate of distance (eccentricity) vs. direction (azimuth vs. elevation) would result in a radial pattern of variable error eigenvectors (noise in the polar representation of distance and direction). Conversely, an independent estimate of target distance and direction would lead to an increase in variability in the *X* or in the *Y* direction, and cause variable errors to align gradually with the *X* or the *Y*-axis, respectively.

#### Accuracy (CE)

In the absence of conflict between the visual and auditory stimuli, the bimodal VA accuracy will be equivalent to the most precise modality, i.e., vision. However, based on the expected differences in precision for A and V in the center and in the periphery, we expected that the contribution of vision in the periphery will be reduced and that of audition increased, due to the predicted reduced gap between visual and auditory precision in this region. For direction, given the fact that A accuracy was greater in the upper than in the lower hemifield, it was expected that the differences in accuracy between A and V in the upper hemifield would be minimal, while remaining substantial in the lower hemifield.

## Materials and methods

### Participants

Three women and seven men, aged 22–50 years, participated in the experiment. They included two of the authors (*MGC* and *PMBS*). All participants possessed a minimum of 20/20 visual acuity (corrected, if necessary) and normal audiometric capacities, allowing for typical age-related differences. They were informed of the overall nature of the experiment. With the exception of the authors, they were unaware of the hypotheses being tested and the details of the stimulus configuration to which they would be exposed.

This study was carried out in accordance with the recommendations of the French Comite Consultatif de Protection des Personnes dans la Recherche Biomédicale (CPPPRB) Paris Cochin and received approval from the CPPPRB. All subjects gave written informed consent in accordance with the Declaration of Helsinki.

### Apparatus

**Figure 1:**
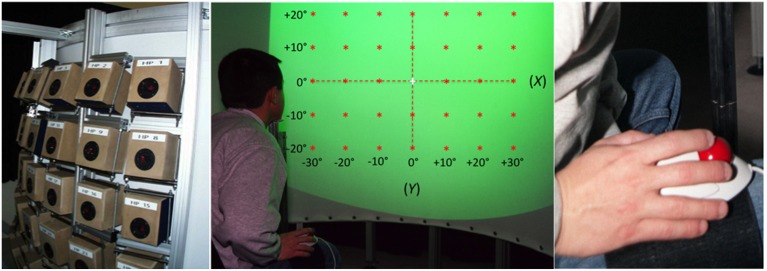
**Experimental setup. Left:** the 35 loudspeakers arranged in a 7 × 5 matrix, with a 10° separation between adjacent speakers both in azimuth and in elevation. **Center:** a participant, head position restrained by a chinrest, is facing the acoustically transparent semi-cylindrical screen. The green area represents the 80° by 60° surface of projection. Red stars depict the location of the 35 targets (±30° azimuth, ±20° in elevation). Note that the reference axes represented here are not visible during the experiment. **Right:** the leg-mounted trackball is attached to the leg of the participant using Velcro straps.

The experimental apparatus (Figure 1) was similar to that used in an earlier study by Godfroy (Godfroy et al., 2003). The participant sat in a chair, head position restrained by a chinrest in front of a vertical, semi-circular screen with a radius of 120 cm and height of 145 cm. The distance between the participant's eyes and the screen was 120 cm. A liquid crystal Phillips Hover SV10 video-projector located above and behind the participant, 245 cm from the screen, projected visual stimuli that covered a frontal range of 80° in azimuth and 60° in elevation (Figure 1, center). The screen was acoustically transparent and served as a surface upon which to project the visual stimuli, which included VA targets, a fixation cross, and a virtual response pointer (a 1°-diameter cross) referenced to as an exocentric technique. Sounds were presented via an array of 35 loudspeakers (10 cm diameter Fostex FE103 Sigma) located directly behind (<5 cm) the screen in a 7 × 5 matrix, with a 10° separation between adjacent speakers in both azimuth and elevation (Figure 1, left). They were not visible to the participant and their orientation was designed to create a virtual sphere centered on the observer's head at eye level.

### The targets

The V target was a spot of light (1° of visual angle) with a luminance of 20 cd/m^2^ (background ca. 1.5 cd/m^2^) presented for 100 ms. The A target was a 100 ms burst of pink noise (broadband noise with constant intensity per octave) that had a 20 ms rise and fall time (to avoid abrupt onset and offset effects) and a 60-ms plateau (broadband sounds have been shown to be highly localizable and less biased, Blauert, 1983). The stimulus duration of 100 ms was chosen based on evidence that auditory targets with durations below 80 ms are poorly localized in the vertical dimension (Hofman and Van Opstal, 1998). The stimulus A-weighted sound pressure level was calibrated to 49 dB using a precision integrating sound level meter (Brüel and Kjäer Model 2230) at the location of the participant's ear (the relative intensity of the A and V stimuli was tested by a subjective equalization test with three participants). The average background noise level (generated by the video-projector) was 38 dB.

Each light spot was projected to the exact center of its corresponding loudspeaker and thus the simultaneous activation and deactivation of the two stimuli created a spatially and temporally congruent VA target. The 35 speakers and their associated light spots were positioned along the azimuth at 0°, ± 10°, ± 20°, and ± 30° (positive rightward) from the SMP and along the vertical dimension at 0°, ± 10°, and ± 20° (positive upward) relative to the HMP. The locations of the V, A, and VA targets are depicted in Figure 1, center.

### Procedure

The participants performed a pointing task to remembered A, V, and AV targets in each of the 35 target locations distributed over the 80 by 60° Frontal field. The participants' task was to indicate the perceived location of the V, A, and VA targets in each of their possible 35 positions by directing a visual pointer to the apparent location of the stimulus via a leg-worn computer trackball, as seen in Figure 1. Besides providing an absolute rather than a relative measure of egocentric location, the advantage of this procedure over those in which the hand, head, or eyes are directed at the targets is that it avoids both (a) the confounding of the mental transformations of sensory target location with the efferent and/or proprioceptive information from the motor system and (b) potential distortions from the use of body-centered coordinates (Brungart et al., 2000; Seeber, 2003).

Prior to each session the chair and the chinrest were adjusted to align participant's head and eyes with the HMP and SMP. After initial instruction and practice, the test trials were initiated each beginning with the presentation of the fixation-cross at the center (0°, 0°) of the semicircular screen for a random period of 500–1500 ms. The participants were instructed to fixate on the cross until its extinction. Simultaneous with the offset of the fixation cross, the V, A, or VA target (randomized) appeared for 100 ms at one of its 35 potential locations (randomized). Immediately following target offset, a visual pointer appeared off to one side of the target in a random direction (0–360°) and by a random amount (2.5–10° of visual angle). The participant was instructed to move the pointer, using a leg-mounted trackball, to the perceived target location (see Figure 1, right). Because the target was extinguished before the localization response was initiated, participants received no visual feedback about their performance. After directing the pointer to the remembered location of the target, the participant validated the response by a click of the mouse, which terminated the trial and launched the next after a 1500 ms interval. The *x*/*y* coordinates of the pointer position (defined as the position of the pointer at the termination of the pointing movement) were registered with a spatial resolution of 0.05 arcmin. Data were obtained from 1050 trials (10 repetitions of each of the 3 modalities × 35 target positions = 1050) distributed over 6 experimental sessions of 175 trials each.

#### The measures of precision and accuracy

The raw data consisted of the 2D coordinates of the terminal position of the pointer relative to a given V, A, or VA target. Outliers (± 3 SD from the mean) were removed for each target location, each modality and each subject to control for intra-individual variability (0.9% for the A condition, 1.3% for the V condition, and 1.4% for the VA condition). To test the hypothesis of colinearity between the *x* and *y* components of the localization responses, a hierarchical multiple regression analysis was performed. Tests for multicollinearity indicated that a very low level of multicollinearity was present [variance inflation factor (*VIF*) = 1 for the 3 conditions]. Results of the regression analysis provided confirmation that the data were governed by a bivariate normal distribution (i.e., 2 dimensions were observed).

To analyze the endpoint distributions, we determined for each target and each modality the covariance matrix of all the 2D responses (*x* and *y* components). The 2D variance ($\sigma_{xy}^{2}$) represents the sum of the variances in the two orthogonal directions ($\sigma_{xy}^{2}=\sigma_{x}^{2}+\sigma_{y}^{2}$). The distributions were visualized by 95% confidence ellipses. We calculated ellipse orientation (θ_*a*_) as the orientation of the main eigenvector (*a*), which represents the direction of maximal dispersion. The *orientation deviations* were calculated as the difference between the ellipse orientation and the direction of the target. Because, an axis is an undirected line where there is no reason to distinguish one end of the line from the other, the data were computed within a 0–180° range. A measure of *anisotropy* of the distributions, ε, was provided, a ratio value close to 1 indicating no preferred direction, and a ratio value close to 0 indicating a preferred direction:

(4) $$\;\varepsilon =\sqrt{1-{\left(b/a\right)}^{2}}$$

For the measure of localization accuracy, the difference between the actual 2D target position and the centroid of the distributions was computed, providing an error vector $\vec{a}$ (Zwiers et al., 2001) that can be analyzed along its length (or amplitude, *r*) and angular direction (α). The mean direction of the error vectors was compared to the target direction, providing a measure of the *direction deviation*. In this study, we assumed that (1) all the target positions were equally likely (the participants had no prior assumption regarding the number and spatial configuration of the targets and (2) the noise corrupting the visual signal was independent from the one corrupting the auditory signal. The present data being governed by a 2D normal distribution, we used a method described previously by Van Beers (Van Beers et al., 1999), which takes into account the “direction” of the 2D distribution. According to Winer (Winer et al., 1991), a 2D normal distribution can be written as:

(5) $$\begin{array}{l} P\left(x,y\right)dxdy=\frac{1}{2\pi \sigma_{x}\sigma_{y}\sqrt{1-\rho^{2}}}exp[-\frac{1}{2\left(1-\rho^{2}\right)}\left(\frac{{\left(x-\mu_{x}\right)}^{2}}{\sigma_{x}^{2}}\right)\;\\ \;+\frac{{\left(y-\mu_{y}\right)}^{2}}{\sigma_{y}^{2}}-\frac{2p\left(x-\mu_{x}\right)\left(y-\mu_{y}\right)}{\sigma_{x}\sigma_{y}}]dxdy \end{array}$$

where $\sigma_{x}^{2}$ and $\sigma_{y}^{2}$ are the variances in the orthogonal *x* and *y* directions, μ_*x*_ and μ_*y*_ are the means in the *x* and *y* directions, and ρ is the correlation coefficient. The parameters of the bimodal VA distribution *P*_*VA*_(*x, y*), i.e., $\sigma_{xVA}^{2}$, $\sigma_{yVA}^{2}$, μ_*xVA*_, and μ_*yVA*_ were computed according to the equations in Appendix 1. The bimodal variance $\left(\sigma_{xyVA}^{2}\right)$, the estimated variance ($\hat{\sigma_{xyVA}^{2}}$), error vectors amplitude (*r*) and direction (α) for each condition were then derived from the initial parameters.

Last we provided a measure of multisensory integration (MSI) by calculating the redundancy gain (*RG*, Charbonneau et al., 2013), assuming vision to be the more effective unisensory stimulus:

(6) $$RG=\left(\frac{\sigma_{xyVA}^{2}}{\sigma_{xyV}^{2}}\right)\times 100$$

Specifically, this measure relates the magnitude of the response to the multisensory stimulus to that evoked by the more effective of the two modality-specific stimulus components. According to the principle of inverse effectiveness (*IE*, Stein and Meredith, 1993), the reliability of the best sensory estimate and *RG* are inversely correlated, i.e., the less reliable single stimulus is associated to maximal *RG* when adding another stimulus.

### The statistical analyses

To allow for comparison between directions, targets located at ±30° eccentricity in azimuth were disregarded. Univariate and repeated measures analyses of variance (ANOVAs) were used to test for the effects of modality (A, V, VA, MLE), direction [*X* (azimuth = horizontal), *Y* (elevation = vertical)] and absolute eccentricity value (0, 10, 14, 20, 22, and 28°). Two-tailed *t*-tests were conducted with Fisher's PLSD (for univariate analyses) and with the Bonferroni/Dunn correction (for repeated measures) for exploring promising *ad hoc* target groupings. These included the comparison between lower hemifield, HMP and upper hemifield on one hand, and left hemifield, SMP and right hemifield on the other hand. Simple and multiple linear regressions were used to determine the performance predictors.

For the measures of the angular/vectorial data [ellipse mean main orientation (θ_*a*_) and vector mean direction (α)], linear regressions were used to assess the fit with the 24 targets orientation/direction [the responses associated to the (0°, 0°) target was excluded since it has, by definition, no direction]. The difference between target and response orientation/direction were computed, allowing for repeated measures between conditions. All of the effects described here were statistically significant at *p* < 0.05 or better.

## Results

### Unimodal auditory and visual localization performance

**Figure 2:**
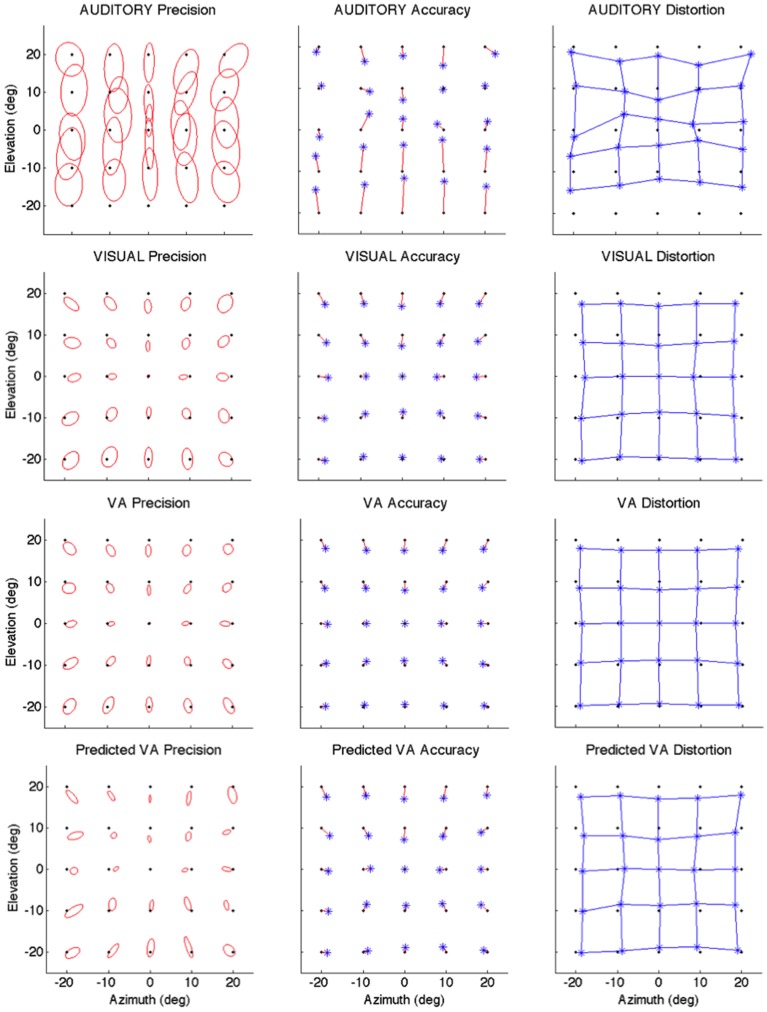
**Localization Precision (left), Accuracy (center) and Local Distortion (right) for the three modalities of presentation of the targets [top to bottom: Auditory, Visual, Visual-Auditory, and predicted VA (MLE)]**. The precision for each of the 25 target positions is depicted by confidence ellipses with the maximum eigenvector (*a*) representing the direction of maximal dispersion. Accuracy: stars represent each of the 25 response centroids linked to its respective target, illustrating the main direction and length of the error vector. Local Distortion: response centroids from adjacent targets are linked to provide a visualization of the fidelity with which the relative spatial organization of the targets is maintained in the configuration of the final pointing positions.

The local characteristics of the local A and V precision, accuracy and distortion are illustrated in Figure 2 and summarized in Table 1.

**Table 1:** **Characteristics of observed A, V, VA, and predicted (MLE) measures of localization precision and accuracy (mean = μ, *sd* = σ)**.

	**A**	**V**	**VA**	**MLE**	**A**	**V**	**VA**	**MLE**
	**μ (σ)**	**μ (σ)**	**μ (σ)**	**μ (σ)**	**μ (σ)**	**μ (σ)**	**μ (σ)**	**μ (σ)**
	**Variable error (precision)**				**Constant error (accuracy)**			
Total (*N* = 25)	5.73 (0.79)	1.78 (0.50)	1.46 (0.37)	1.53 (0.36)	4.03 (2.37)	2.00 (0.87)	1.67 (0.72)	1.94 (0.69)
	**Orientation deviation**				**Direction deviation**			
Total (*N* = 25)	39.14 (28.66)	13.05 (11.57)	13.57 (13.52)	25.63 (22.27)	43.02 (27.15)	16.74 (15.91)	12.74 (11.59)	16.52 (14.15)

#### Auditory

**Figure 3:**
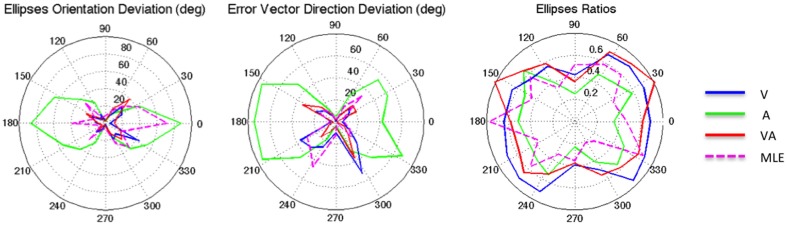
**From left to right: Ellipse Orientation Deviation, Error Vector Orientation Deviation, and Ellipse Ratio in the polar coordinate system**.

It can be seen from Figure 2 that auditory localization was characterized by anisotropic response distributions oriented upward over the entire field. The difference in orientation between the target and the ellipse main orientation was highest in azimuth and lowest in elevation (*X*: μ = 86.83°, *sd* = 2.40; *Y*: μ = 1.93°, *sd* = 0.57; *X,Y*: *t* = 84.89, *p* < 0.0001, see Figure 3, left). These scatter properties emphasize the fact that azimuth and elevation localization are dissociate processes (see Introduction). Note also that the ellipses were narrower in the SMP than elsewhere (ε: SMP = 0.23; periphery = 0.50; SMP, periphery: *t* = −0.26, *p* < 0.0001), as seen in Figures 2, 3, right. Auditory localization precision was statistically equivalent in the *X* and *Y* direction (*X*: μ = 5.52, *sd* = 0.72; *Y*: μ = 5.34, *sd* = 1.26; *X*,*Y*: *t* = 0.17, *p* = 0.76). There was no significant effect of eccentricity [*X: F*_(5, 19)_ = 0.70, *p* = 0.62].

**Figure 4:**
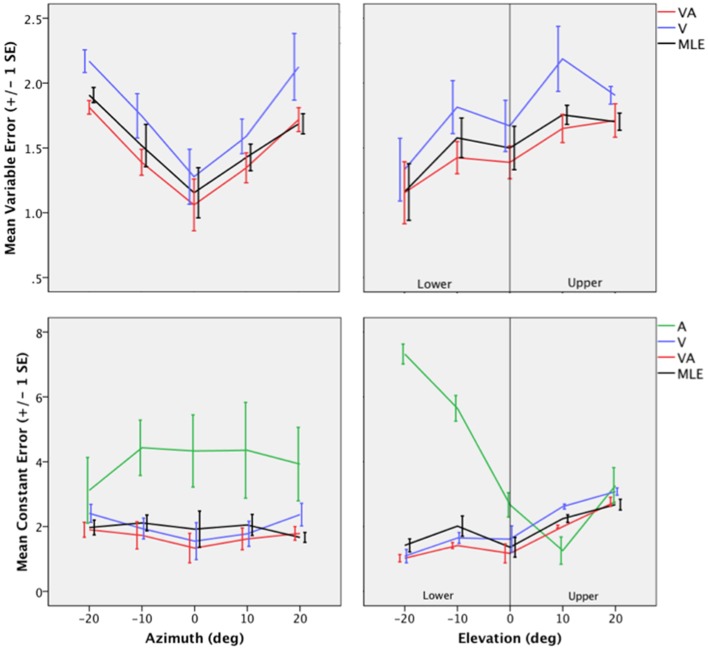
**Top:** Mean Variable Error (*VE*) for the V, VA, and the MLE as a function of eccentricity in Azimuth (left) and eccentricity in Elevation (right). **Bottom:** Mean Constant Error (*CE*) for the A, V, VA conditions and the MLE as a function of eccentricity in Azimuth (left) and eccentricity in Elevation (right).

**Figure 5:**
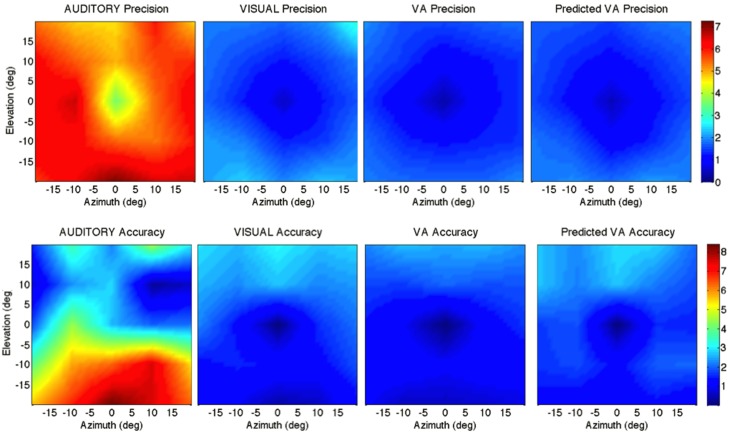
**Top:** precision across the 2D frontal field (horizontal axis = −20°, +20°; vertical axis = −20°, +20°). From left to right: A, V, VA and MLE (predicted VA). The color bar depicts the precision in localization from extremely precise (blue) to imprecise (red). **Bottom:** accuracy across the 2D frontal field (horizontal axis = −20°, +20°; vertical axis = −20°, +20°). From left to right: A, V, VA, and MLE. The color bar depicts localization accuracy from more accurate (blue) to less accurate (red). Auditory localization was more accurate in the upper than in the lower hemifield while the opposite holds true for visual localization.

Auditory localization accuracy was characterized by significant undershoot of the responses in elevation, as seen in Figures 2, 3, center, where the error vector directions are opposite to the direction of the targets relative to the initial fixation point. Auditory localization was more accurate by a factor of 3 in the upper hemifield than in the lower hemifield (upper: μ = 2.26°, *sd* = 1.47; lower: μ = 6.48°, *sd* = 1.15; upper, lower: *t* = −4.22, *p* < 0.0001), resulting in an asymmetrical space compression (see Figures 2, 4, 5). The highest accuracy was observed for targets 10° above the HMP (*Y* = 0°: μ = 2.66, *sd* = 0.83; *Y* = +10°: μ = 1.25, *sd* = 0.94; 0°,+10°: *t* = 1.41, *p* = 0.02), suggesting that the A and the V “horizons” may not coincide, as was reported, though not discussed, by Carlile (Carlile et al., 1997). There was no effect of eccentricity in azimuth [*F*_(2, 22)_ = 0.36, *p* = 0.69].

#### Visual

The topology of the visual space was characterized by a radial pattern of the errors in all directions, as seen in Figure 2, where all the variance ellipses are aligned in the direction of the targets, relative to the initial fixation point [regression target/ellipse orientation: *R*^2^ = 0.89, *F*_(1, 22)_= 205.28, *p* < 0.0001; *r* = 0.95, *p* < 0.0001]. The ellipses were narrower in the SMP than in the HMP, differences that were statistically significant (ε: SMP = 0.41; HMP = 0.63; SMP, HMP: *t* = 0.22, *p* = 0.001). For targets of the two orthogonal axes, the ratio was statistically equivalent to that in the *X* axis direction (see Figure 3, right). The overall orientation deviation was independent of the target direction (*X*: μ = 9.12°, *sd* = 6.75; *Y*: μ = 2.45°, *sd* = 2.23; *X*,*Y*: *t* = 6.66, *p* = 0.39), as seen in Figure 3, left. These scatter properties reveal the polar organization of the visuomotor system (Van Opstal and Van Gisbergen, 1989). The VA localization was slightly more precise in elevation than in azimuth, although the difference didn't quite reach significance (*X*: μ = 1.77, *sd* = 0.42; *Y*: μ = 1.29, *sd* = 0.54; *X, Y*: *t* = 0.49, *p* = 0.09). Precision decreased systematically with eccentricity in azimuth [*F*_(2, 22)_ = 8.88, *p* = 0.001], but not in elevation [*F*_(2, 22)_ = 1.67, *p* = 0.21], as seen in Figures 4, 5, where one can see that the variability was higher in the upper hemifield than in the lower hemifield (upper: μ = 2.04, *sd* = 0.41; lower: μ = 1.57, *sd* = 0.53; upper, lower: *t* = 0.47, *p* = 0.03).

Visual accuracy was characterized by a systematic undershoot of the responses, i.e., the vectors direction was opposite to the direction of the target, and the difference between target and vector direction averaged 180° over the entire field (direction deviation: μ = 165.04°, *sd* = 47.64). The direction deviations were marginally larger for targets with an oblique direction (i.e., 45, 135, 225, and 315° directions) than for targets on the two orthogonal axes (*X*,*XY*: *t* = −17.96, *p* = 0.06; *Y*,*XY*: *t* = −17.46, *p* = 0.07, see Figure 3, center). The localization bias (*CE*) represented 11.9% of the target eccentricity, a value that conforms to previous studies, and was consistent throughout directions and eccentricities. Note that the compression of the visual space, resulting from the target undershoot, was more pronounced in upper hemifield than in the lower hemifield (upper: μ = 2.84, *sd* = 0.31; lower: μ = 1.36, *sd* = 0.49; upper, lower: *t* = 1.47, *p* < 0.0001, see Figures 2, 4, 5), an effect opposite to that observed for A localization accuracy.

### Bimodal visual-auditory localization performance

#### Observed

The response distributions showed anisotropic distributions with the main eigenvector oriented in the direction of the targets relative to the initial fixation point [regression target/ellipse orientation: *R*^2^ = 0.87, *F*_(1, 22)_ = 158.37, *p* < 0.0001; *r* = 0.93, *p* < 0.0001] as seen in Figures 2, 3. As previously reported in the A and the V conditions, the ellipse distributions were narrower in the SMP than in the HMP (ε: SMP = 0.37; HMP = 0.55; SMP, HMP: *t* = 0.18, *p* = 0.01). The overall orientation deviation was independent of the target direction (*X*: μ = 9.04°, *sd* = 3.83; *Y*: μ = 3.23°, *sd* = 2.80; *X*,*Y*: *t* = 5.81, *p* = 0.52).

The VA localization was marginally more precise in elevation than in azimuth (*X*: μ = 1.49, *sd* = 0.18; *Y*: μ = 1.08, *sd* = 0.51; *X*,*Y*: *t* = 0.41, *p* = 0.07), and decreased systematically with eccentricity in azimuth [*F*_(2, 22)_ = 13.13, *p* < 0.0001], but not in elevation [*F*_(2, 22)_ = 0.31, *p* = 0.73]. However, the variability was higher in the upper hemifield than in the lower hemifield (upper: μ = 1.68, *sd* = 0.25; lower: μ = 1.28, *sd* = 0.24; upper, lower: *t* = 0.39, *p* = 0.01), a characteristic previously reported for visual precision.

The direction deviations were on average four times larger for targets with an oblique direction than for targets in the two orthogonal axes (*X*: μ = 2.40, *sd* = 1.67; *Y*: μ = 3.42, *sd* = 3.74; *XY*: μ = 18.76, *sd* = 10.29; *X*,*Y*: *t* = −1.02, *p* = 0.88; *X*,*XY*: *t* = −16.36, *p* = 0.01; *Y*,*XY*: *t* = −15.33, *p* = 0.02). As for vision, VA localization showed a systematic target undershoot in all directions, as illustrated in Figures 2, 3, where one can see that the direction of the vectors is opposite to the direction of the target. The localization bias (μ = 1.39, *sd* = 0.65) represented 9.22% of the target eccentricity, a value that decreased slightly with eccentricity without reaching significance [*F*_(3, 12)_ = 3.17, *p* = 0.06]. There was no effect of direction. Bimodal accuracy was not affected by the effect of direction (*X*: μ = 1.43, *sd* = 0.32; *Y*: μ = 1.64, *sd* = 0.87; *X*,*Y*: *t* = −0.20, *p* = 0.68) and decreased slightly with eccentricity [*F*_(5, 19)_ = 1.40, *p* = 0.26]. One may observe that VA accuracy was highest in the lower than in the upper hemifield (upper, lower: *t* = 1.16, *p* < 0.0001), a characteristic already shown for visual localization accuracy (see Figures 2, 4, 5). In the upper hemifield, the magnitude of undershoot averaged 2.38 ± 0.45°, which is almost twice as much as what was observed in the lower hemifield (1.21 ± 0.30°).

#### Predicted

The model predicted anisotropic response distributions, with in general the main eigenvector aligned with the direction of the target relative to the initial fixation point (regression target/ellipse orientation: *R*^2^ = 0.38, *F*_(1, 22)_ = 13.71, *p* = 0.01]. Interestingly, the MLE didn't predict variations in the anisotropy of the distributions as a function of direction (ε: SMP = 0.43; HMP = 0.58; SMP, HMP: *t* = 0.14, *p* = 0.29). The orientation deviation was larger in azimuth than in elevation (*X*: μ = 47.19°, *sd* = 34.62; *Y*: μ = 7.57°, *sd* = 5.90; *X, Y*: *t* = 39.61, *p* = 0.01), as seen in Figure 3, left. The predicted variance was statistically equivalent in the *X* and *Y* directions (*X*: μ = 1.58, *sd* = 0.37; *Y*: μ = 1.15, *sd* = 0.50; *X, Y*: *t* = 0.43, *p* = 0.06). The effect of eccentricity was significant in azimuth [*F*_(2, 22)_ = 8.72, *p* = 0.002] but not in elevation [*F*_(2, 22)_ = 1.05, *p* = 0.36] but the variance was higher in the upper hemifield than in the lower hemifield (upper: μ = 1.72, *sd* = 0.15; lower: μ = 1.36, *sd* = 0.45; upper, lower: *t* = 0.35, *p* = 0.02; see Figures 2, 4, 5).

Vector direction deviations were larger in the oblique direction than in the orthogonal directions, as seen in Figure 3, center (*X*: μ = 6.27, *sd* = 5.62; *Y*: μ = 6.95, *sd* = 7.08; *XY*: μ = 25.33, *sd* = 14.90; *X*,*Y*: *t* = −0.67, *p* = 0.94; *X*,*XY*: *t* = −19.06, *p* = 0.02; *Y*,*XY*: *t* = −18.38, *p* = 0.03). The predicted accuracy showed a systematic target undershoot in all directions, as illustrated in Figures 2, 3, where one can see that the direction of the vectors is opposite to the direction of the target. The localization bias (μ = 1.80, *sd* = 0.67) represented 10.85% of the target eccentricity, a value that decreased with eccentricity [*F*_(4, 19)_ = 8.43, *p* < 0.0001]. There was no effect of direction (*X, Y*: *t* = −0.35, *p* = 0.43) or eccentricity [*F*_(5, 19)_ = 1.72, *p* = 0.17]. The difference in accuracy between upper and lower hemifield observed in the VA condition was well-predicted (upper, lower: *t* = 0.74, *p* = 0.003), with an undershoot magnitude of 2.46 ± 0.37° in the upper hemifield and 1.71 ± 0.63° in the lower hemifield (see Figures 4, 5).

### Applying the MLE model to the VA localization and accuracy

#### Orientation deviation

The magnitude of the ellipses orientation deviation (ellipse orientation in relation to the target direction) was very similar in the V and in the VA condition (V: μ = 13.05°, *sd* = 2.36°; VA: μ = 13.67°, *sd* = 2.76°; *t* = 0.48, *p* = 1), as seen in Figure 3, where the plots for V and VA almost overlap. The MLE predicted larger orientation deviations than observed in the VA condition (μ = 24.73°, *sd* = 22.58°, VA, MLE: *t* = −12.68, *p* = 0.007), primarily in the *Y* and *XY* directions.

#### Precision

Figure 5 top depicts from left to right, the 2D variance ($\sigma_{XY}^{2}$) for the A, V, VA targets and the predicted MLE estimate. It illustrates the inter- and intra-modality similarities and differences reported earlier. Note the left/right symmetry for all conditions, the greater precision for audition in the upper hemifield than in the lower hemifield and the improved precision in the VA condition compared to the V condition. The ellipse ratio was higher (i.e., ellipses less anisotropic) in the observed VA condition than in the predicted VA condition (ε: VA=.60; MLE=.48; VA, MLE: *t* = 0.11, *p* = 0.002), potentially as a result of an expected greater influence of audition. Comparison between the V, VA and MLE conditions showed a significant effect of modality [*F*_(2, 48)_ = 24.71, *p* < 0.0001], with less variance in the VA condition than in the V condition (V, VA: *t* = 0.31, *p* < 0.0001). There was no difference between observed and predicted precision (*t* = −0.07, *p* = 0.16). There was no interaction with direction [*F*_(2, 12)_ = 0.34, *p* = 0.71], eccentricity [*F*_(10, 38)_ = 1.33, *p* = 0.24] or upper/lower hemifield [*F*_(2, 36)_ = 0.53, *p* = 0.59].

VA precision was significantly correlated with both A and V precision ($\sigma_{xyA}^{2},\sigma_{xyAV}^{2}$: *r* = 0.46, *p* = 0.01; $\sigma_{xyV}^{2},\sigma_{xyAV}^{2}$: *r* = 0.82, *p* < 0.0001), which was well-predicted by the model ($\sigma_{xyA}^{2},\hat{\sigma_{xyVA}^{2}}:$ *r* = 0.57, *p* = 0.002; $\sigma_{xyV}^{2},\hat{\sigma_{xyVA}^{2}}$: *r* = 0.91, *p* < 0.0001; $\sigma_{xyAV}^{2},\hat{\sigma_{xyVA}^{2}}$: *r* = 0.88, *p* < 0.0001).

**Figure 6:**
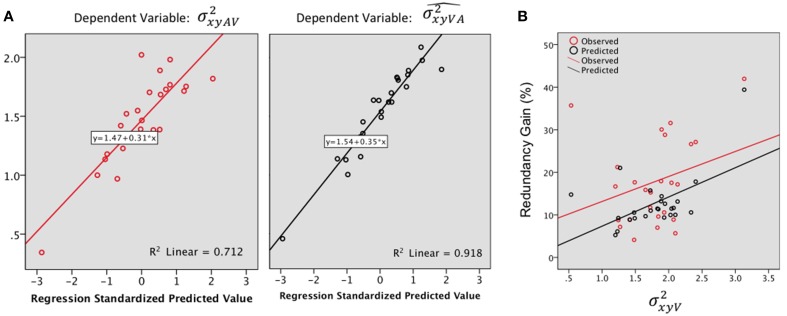
**(A)** Regression plots for the bimodal observed ($\sigma_{xyAV}^{2}$, left) and predicted variance ($\hat{\sigma_{xyVA}^{2}}$, right). Predictors: $\sigma_{xyV}^{2},\sigma_{xyA}^{2}$. **(B)** Redundancy gain (*RG*, in %) as a function of the magnitude of the variance in the visual condition ($\sigma_{xyV}^{2}$). The *RG* increases as the reliability of the visual estimate decreases (variance increases). Note that the model prediction parallels the observed data, although the magnitude of the observed *RG* was significantly higher than predicted by the model.

Step by step linear regressions (method Enter) were performed to assess the contribution of V and A precision as predictors of the observed and predicted VA localization precision. In the observed VA condition (Figure 6A, Left), 68% of the variance was explained, exclusively by $\sigma_{xyV}^{2}$ [(Constant), $\sigma_{xyV}^{2}$: *R*^2^= 0.67; adjusted *R*^2^ = 0.66; *R*^2^ change = 0.67; *F*_(1, 23)_ = 47.69, *p* < 0.0001; (Constant), $\sigma_{xyV}^{2},\sigma_{xyA}^{2}$: *R*^2^ = 0.71; adjusted *R*^2^ = 0.68; *R*^2^ change = 0.03; *F*_(1, 22)_ = 2.85, *p* = 0.1]. Conversely, the model predicted a significant contribution of both the A and the V precision with an adjusted *R*^2^ of 0.91; i.e., 91% of the total variance was explained [see Figure 6A right, (Constant), $\sigma_{xyV}^{2}$: *R*^2^ = 0.84; adjusted *R*^2^= 0.83; *R*^2^ change = 0.84; *F*_(1, 23)_ = 122.83, *p* < 0.0001; (Constant), $\sigma_{xyV}^{2},\sigma_{xyA}^{2}$: *R*^2^ = 0.91; adjusted *R*^2^ = 0.91; *R*^2^ change = 0.07; *F*_(1, 22)_=20.39, *p* < 0.0001].

The observed *RG* (18.07%) was positive for 96% (24) of the tested locations and was statistically higher than the model prediction (12.76%) [*F*_(1, 23)_ = 7.98, *p* = 0.01]. There was no significant difference in gain, observed or predicted, throughout main direction and eccentricity.

In order to further investigate the association between the *RG* and unimodal localization precision, we correlated the RG with the mean precision for the best unisensory modality. The highest observed RG were associated with the less precise unimodal estimate (Figure 6B), although the correlation didn't quite reach significance (Pearson's *r* = 0.29, *p* = 0.07). Meanwhile, the model predicted well the *IE* effect (Figure 6B) with a significant correlation between RG and visual variance (Pearson's *r* = 0.53, *p* = 0.004).

#### Direction deviation

The magnitude of the vector direction deviation was statistically equivalent between V, VA, and MLE [*F*_(246)_ = 1.36, *p* = 0.25]. In both conditions, the orientation deviations were larger for targets with an oblique direction than on the two orthogonal axes (i.e., around the 45, 135, 225, and 315° directions).

#### Accuracy

Comparison between V and VA accuracy showed that VA accuracy was not an intermediate between the A and the V accuracy and that overall, the AV responses were more accurate than in the V condition (*r*_*V*_, *r*_*VA*_: *t* = 0.33, *p* < 0.0001). Conversely, accuracy predicted by the model was not statistically different than in the V condition (*r*_*V*_, $\hat{r_{VA}}$: *t* = 0.06, *p* = 0.62; *r*_*VA*_, $\hat{r_{VA}}$: *t* = −0.26, *p* = 0.01) while statistically different than observed (*r*_*VA*_, $\hat{r_{VA}}$: *t* = −4.98, *p* < 0.0001). There was no significant effect of interaction with direction [*F*_(15, 57)_ = 0.66, *p* = 0.81] or eccentricity [*F*_(15, 57)_ = 0.14, *p* = 1]. These general observations obscured local differences between modalities. Indeed, there was a significant effect of interaction between modality and upper/lower hemifield [*F*_(6, 66)_ = 34.56, *p* < 0.0001] as seen in Figures 4, 5. A first relatively unexpected result is the fact A and V accuracy were not statistically different in the upper hemifield (*r*_*A*_: μ = 2.26, *sd* = 1.47; *r*_*V*_: μ = 2.84, *sd* = 0.31; *r*_*A*_, *r*_*V*_: *t* = −1.31, *p* = 0.22), although some local differences in the periphery are visible from Figure 5. Conversely, in the lower hemifield, V localization was on average more accurate by an order of 5 than A localization (*r*_*A*_: μ = 6.48, *sd* = 1.15; *r*_*V*_: μ = 1.36, *sd* = 0.49; *r*_*A*_, *r*_*V*_: *t* = 5.11, *p* < 0.0001). These differences between unimodal conditions provide a unique opportunity to evaluate the relative contribution of A and V to the bimodal localization performance.

In the upper hemifield, the VA localization was more accurate than in the V condition (*r*_*V*_, *r*_*VA*_: *t* = 3.85, *p* = 0.004), but not than in the A condition (*r*_*A*_, *r*_*VA*_: *t* = −0.31, *p* = 0.76). The model also predicted this pattern (*r*_*A*_, $\hat{r_{VA}}$: *t* = −0.49, *p* = 0.63; *r*_*V*_, $\hat{r_{VA}}$: *t* = 2.66, *p* = 0.02), and therefore, the difference between observed and predicted accuracy was not significant (*r*_*VA*_, $\hat{r_{VA}}$: *t* = −0.74, *p* = 0.47).

In the lower hemifield, however, V and VA accuracy localization was not statistically different (*r*_*V*_, *r*_*VA*_: *t* = −1.83, *p* = 0.10). Meanwhile, the accuracy predicted by the model (μ = 1.71, *sd* = 0.63), less homogeneous, was not different from the V condition (*r*_*V*_, $\hat{r_{VA}}$: *t* = −1.47, *p* = 0.17), but the predicted VA localization was significantly less accurate than observed (*r*_*VA*_, $\hat{r_{VA}}$: *t* = −2.30, *p* = 0.04).

#### Relationships between precision and accuracy

According to the MLE, the VA accuracy depends, at various levels, upon the unimodal A and V precision. The visual weight (*W*_*V*_) was computed to provide an estimate of the respective unimodal contribution as a function of direction and eccentricity.

**Figure 7:**
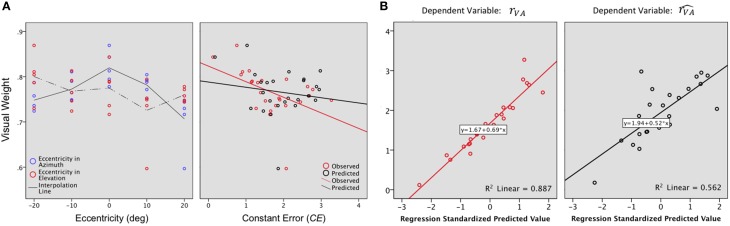
**(A)** Visual weight. A value of 0.5 would indicate an equivalent contribution of the A and the V modalities to the VA localization precision. For the examined region (−20 to +20° azimuth, −20 to +20° azimuth), *W*_*V*_ values were 0.60 to 0.90, indicating that vision always contributed more than audition to bimodal precision. Left: In azimuth, *W*_*V*_ decreases as the eccentricity of the target increases. In elevation, *W*_*V*_ was marginally higher in the lower than in the upper hemifield. Right: VA accuracy is inversely correlated to *W*_*V*_ i.e., the highest values of *W*_*V*_ were associated with the smallest *CEs*. **(B)** Regression plots for the bimodal observed (*r*_*VA*_, left) and predicted accuracy ($\hat{r_{VA}}$, right). Significant predictors: *W*_*V*_, *r*_*A*_ and *r*_*V*_ for *r*_*VA*_; *r*_*V*_ for $\hat{r_{VA}}$.

Vision, which is the most reliable modality for elevation, was expected to be associated with a stronger weight along the elevation axis than along the azimuth axis. This is indeed what was observed (*W*_*V*_: *X*: μ = 0.75, *sd* = 0.03; *W*_*V*_: *Y*: μ = 0.81, *sd* = 0.03; *X*,*Y*: *t* = −0.05 *p* = 0.05). As expected, the visual weight decreased significantly with eccentricity in azimuth [*F*_(2, 22)_ = 10.25, *p* = 0.001] but not in elevation [*F*_(2, 22)_ = 1.16, *p* = 0.33], as seen in Figure 7A, left. In this axis, *W*_*V*_ was marginally higher in the lower hemifield than in the upper hemifield (upper: μ = 0.74; lower: μ = 0.78; upper, lower: *t* = −0.04, *p* = 0.07).

Overall, VA accuracy was inversely correlated to *W*_*V*_ (*R*_*VA*_,*W*_*V*_: *r* = −0.48, *p* = 0.007), i.e., the highest values of *W*_*V*_ were associated with the smallest values of *CEs*, as seen in Figure 7A, right. However, *W*_*V*_ alone explained only 20% of the total variance, a contribution that was significant [(Constant), *W*_*V*_: *R*^2^ = 0.24; adjusted *R*^2^= 0.20; *R*^2^ change = 0.24; *F*_(1, 32)_ = 7.24, *p* = 0.01]. A step-by-step linear regression was then performed to assess the potential additional contribution of the V and A accuracy to the bimodal accuracy (*R*_*VA*_). Altogether, the three parameters explained 87% of the total variance, with a major contribution of *R*_*V*_ [Figure 7B left (Constant), *W*_*V*_, *R*_*A*_: *R*^2^ = 0.31; adjusted *R*^2^ = 0.24; *R*^2^ change = 0.07; *F*_(1, 22)_ = 2.26, *p* = 0.14; (Constant), *W*_*V*_*,R*_*A*_, *R*_*V*_: *R*^2^ = 0.24; adjusted *R*^2^ = 0.87; *R*^2^ change = 0.57; *F*_(1, 21)_ = 107.47, *p* < 0.0001].

The bimodal VA accuracy was significantly correlated to both V and A accuracy (*r_V_*,*r*_VA:_ *r* = 0.92, *p* < 0.0001; *r_A_,r_VA_ r* = −0.47, *p* = 0.01). Of interest here is the negative correlation between *r*_*A*_ and _*r*_*R*_*V*_ (*r_A_*,*r_V_*: *r* = −0.64, *p* < 0.0001), suggesting a trade-off between A and V accuracy.

Meanwhile, there was no significant correlation between the performance predicted by the MLE and *W*_*V*_ ($\hat{r_{VA}}$,*W*_*V*_: *r* = −0.15, *p* = 0.22) and the 49% of explained variance were attributable exclusively to *r*_*V*_ [Figure 7B right (Constant), *W*_*V*_: *R*^2^ = 0.02; adjusted *R*^2^ = −0.01; *R*^2^ change = 0.02; *F*_(1, 23)_ = 0.58, *p* = 0.45; (Constant), *W*_*V*_*,R*_*A*_: *R*^2^ = 0.06; adjusted *R*^2^ = −0.16; *R*^2^ change = 0.04; *F*_(1, 22)_ = 1.03, *p* = 0.31; (Constant), *W_V_,R_A_, R_V_*: *R*^2^ = 0.56; adjusted *R*^2^ = 0.49; *R*^2^ change = 0.49; *F*_(1, 21)_ = 23.64, *p* < 0.0001].

Because, the bimodal visual-auditory localization was shown to be more accurate than the most accurate unimodal condition, which was not predicted by the model, one may ask whether the bimodal precision could predict bimodal accuracy. Indeed, there was a significant positive correlation between VA precision and VA accuracy ($\sigma_{xyVA}^{2},$*r*_*VA*_: *r* = 0.62, *p* = 0.001), a relation not predicted by the model ($\hat{\sigma_{xyVA}^{2}}$,$\hat{r_{VA}}$: *r* = 0.30, *p* = 0.13).

## Discussion

The present research reaffirmed and extended previous results by demonstrating that the two-dimensional localization performance of spatially and temporally congruent visual-auditory stimuli generally exceeds that of the best unimodal condition, vision. Establishing exactly how visual-auditory integration occurs in the spatial dimension is not trivial. Indeed, the reliability of each sensory modality varies as a function of the stimulus location in space, and second, each sensory modality uses a different format to encode the same properties of the environment. We capitalized on the differences in precision and accuracy between vision and audition as a function of spatial variables, i.e., eccentricity and direction, to assess their respective contribution to bimodal visual-auditory precision and accuracy. By combining two-dimensional quantitative and qualitative measures, we provided an exhaustive description of the performance field for each condition, revealing local and global differences. The well-known characteristics of vision and audition in the frontal perceptive field were verified, providing a solid baseline for the study of visual-auditory localization performance. The experiment yielded the following findings.

First, visual-auditory localization precision exceeded that of the more precise modality, vision and was well-predicted by the MLE. The redundancy gain observed in the bimodal condition, signature of crossmodal integration (Stein and Meredith, 1993) was greater than predicted by the model and supported an inverse effectiveness effect. The magnitude of the redundancy gain was relatively constant regardless the reliability of the best unisensory component, a result previously reported by Charbonneau (Charbonneau et al., 2013) for the localization of spatially congruent visual-auditory stimuli in azimuth. The bimodal precision, both observed and predicted, was positively correlated to the unimodal precision, with a ratio of 3:1 for vision and audition, respectively. Based on the expected differences in precision for A and V in the center and in the periphery, we expected that the contribution of vision in the periphery will be reduced and that of audition increased, due to the predicted reduced gap between visual and auditory precision in this region. For direction, vision, which is the most reliable modality for elevation was given a stronger weight along the elevation axis than along the azimuth axis. Less expected was the fact that the visual weight decreased with eccentricity in azimuth only. In elevation, the visual weight was greater in the lower than in the upper hemifield. Meanwhile, the eigenvector's radial localization pattern supported a polar representation of the bimodal space, with directions similar to those in the visual condition. For the model, the eigenvector's localization pattern supported a hybrid representation, in particular for loci where the orientations of the ellipses between modalities were the most discrepant. One may conclude at this point that the improvement in precision for the bimodal stimulus relative to the visual stimulus revealed the presence of optimal integration well-predicted by the Maximum Likelihood Estimation (MLE) model. Further, the bimodal visual-auditory stimulus location appears to be represented in a polar coordinate system at the initial stages of processing in the brain.

Second, visual-auditory localization was also shown to be, on average, more accurate than visual localization, a phenomenon unpredicted by the model. We observed performance enhancement in 64% of the cases, against 44% for the model. In the absence of spatial discrepancy between the visual and the auditory stimuli, the overall MLE prediction was that the bimodal visual-auditory localization accuracy would be equivalent to the most accurate unimodal condition, vision. The results showed that locally, bimodal visual-auditory localization performance was equivalent to the most accurate unimodal condition, suggesting a *relative* rather than an *absolute* sensory dominance. Of particular interest was how precision was related to accuracy when a bimodal event is perceived as unified in space and time. Overall, VA accuracy was correlated to the visual weight, the stronger the visual weight the greater the VA accuracy. However, visual accuracy was a greater predictor of the bimodal accuracy than the visual weight. Also, our results support some form of transitivity between the performance for precision and accuracy, with 62% of the cases of performance enhancement for precision leading also to performance enhancement for accuracy. As for precision, the magnitude of the redundancy gain was relatively constant regardless the reliability of the best unisensory component. There was no reduction in vector direction deviations in the bimodal condition, which was well-predicted by the model. For all the targets, we observed a relatively homogeneous and proportional underestimation of target distance, with constant errors directed inward toward the origin of the polar coordinate system. The resulting array of the final positions was an undistorted replica of the target array, displaced by a constant error common to all targets. The local distortion (which refers to the fidelity with which the relative spatial organization of the targets is maintained in the configuration of the final pointing positions, McIntyre et al., 2000) indicates an isotropic contraction, possibly produced by an inaccurate sensorimotor transformation.

Lastly, the measurement of the bimodal local distortion represents a local approximation of a global function that can be approximated by a linear transformation from target to endpoint position as presented in Appendix 2. One can see the similarities between the functions that describe visual and bimodal local distortion. Meanwhile, the pattern of parallel constant errors observed in the auditory condition reveal a Cartesian representation. The distortions and discrepancies in auditory and visual space described in our results can find two main explanations. The first is the possibility that open-loop response measures of egocentric location that involve reaching or pointing are susceptible to confounding by motor variables and/or a reliance on body-centric coordinates. For example, it might be proposed that reaching for visual objects is subject to a motor bias that shifts the response toward the middle of the visual (and body-centric) field, resulting in what appears to be a compression of visual space where none actually exists. A second potential concern with most response measures is that because they involve localizing a target that has just been extinguished, their results may apply to memory-stored rather than currently perceived target locations (Seth and Shimojo, 2001). The present results support the fact that short-term-memory distortions may have affected the localization performance. The results also speak against the amodality hypothesis (i.e., spatial images have no trace of their modal origins, Loomis et al., 2012) because the patterns of responses clearly reveal the initial coding of the stimuli.

The major contribution of the present research was the demonstration of how the differences between auditory and visual spatial perception, some of which have been reported previously, relate to the interaction of the two modalities in the localization of the VA targets across the 2D frontal field. First, localization response and accuracy were estimated in two dimensions, rather than being decomposed artificially into separate, non-collinear *x* and *y* response components. Another important difference with previous research is that we used spatially congruent rather than spatially discrepant stimuli, which were both considered optimal for the task. The differences in precision and accuracy for vision and audition were used to create different ecological levels of reliability of the two modalities instead of capitalizing on the artificial degradation of one or the other stimuli. One may argue that the integration effect would have been greater by using degraded stimuli. This is indubitably true, but this may have obscured the role of eccentricity and direction.

Two other important distinctions between the present research and previous similar efforts were the use of (a) “free field” rather than binaurally created auditory targets and (b) an absolute (i.e., egocentric) localization measure (Oldfield and Parker, 1984; Hairston et al., 2003a), rather than a forced-choice (relative) one (Strybel and Fujimoto, 2000; Battaglia et al., 2003; Alais and Burr, 2004). The advantage of using actual auditory targets is that they are known to provide better cues for localization in the vertical dimension than are binaural stimuli (Blauert, 1997) and are, of course, more naturalistic. With respect to the localization measure, although a forced-choice indicator (e.g., “Is the sound to the left of the light or to the right?”) is useful for some experimental questions, it was inappropriate for our research in which the objective was to measure exactly where in 2D space the V, A, and VA targets appeared to be located. For example, although a forced-choice indicator could be used to measure localization accuracy along the azimuth and elevation, it would be insensitive to any departures from these canonical dimensions. For example, it could not discriminate between a sound that was localized 2° to the right of straight ahead along the azimuth from one localized 2° to the right and 1° above the azimuth. Our absolute measure in which participants directed a visual pointer at the apparent location of the target is clearly not constrained in this way.

At this point, it is important to note that the effects reported here could appear quite modest in regards to previous studies. This was expected given the fact we used *non-degraded* and *congruent* visual and auditory stimuli. Increasing the size of the test region, especially in azimuth, would allow modifying even more the relative reliability of vision and audition to the point where audition would dominate vision. Another limit in our study is that we used a head-restrained method that could have contributed to some of the reported local distortions. Combining a wider field and a head-free method would provide the opportunity to investigate spatial visual-auditory interactions in a more ecological framework.

In conclusion, these results demonstrate that spatial locus, i.e., the spatial congruency effect (SCE), must be added to the long list of factors that influence the relative weights of audition and vision for spatial localization. Thus, rather than making the blanket statement that vision dominates audition in spatial perception, it is important to determine the variables that contribute to (or reduce) this general superiority. The present results clearly show that the two-dimensional target's locus is one of these variables. Finally, we would argue that because our research capitalized on naturally occurring spatial discrepancies between vision and audition using ecologically valid stimulus targets rather than laboratory creations, its results are especially applicable to the interaction of these sensory modalities in the everyday world.

### Conflict of interest statement

The Guest Associate Editor Guillaume Andeol declares that, despite sharing an affiliation with the author Patrick Maurice Basile Sandor at the Institut de Recherche Biomédicale des Armées, the review was handled objectively. The authors declare that the research was conducted in the absence of any commercial or financial relationships that could be construed as a potential conflict of interest.

## Appendix 1

$$\begin{array}{l} \sigma_{xVA}^{2}=A/(AB-E^{2})\\ \sigma_{yVA}^{2}=B/(AB-E^{2})\\ \mu_{xVA}=(BC+ED)/(AB-E^{2})\\ \mu_{yVA}=(AD+EC)/(AB-E^{2})\\ \;p_{VA}=E/\sqrt{AB}\\ \;A=\frac{1}{(1-p_{V}^{2})S_{xV}^{2}}+\frac{1}{(1-p_{A}^{2})S_{xA}^{2}}\\ \;B=\frac{1}{(1-p_{V}^{2})\sigma_{yV}^{2}}+\frac{1}{(1-p_{A}^{2})\sigma_{yA}^{2}}\\ \;C=\frac{1}{1-p_{V}^{2}}\left(\frac{\mu_{yV}}{\sigma_{yV}^{2}}-\frac{p_{V\mu_{yV}}}{\sigma_{xV}S_{yV}}\right)+\frac{1}{1-p_{A}^{2}}\left(\frac{\mu_{yA}}{\sigma_{yA}^{2}}-\frac{p_{A\mu_{yA}}}{\sigma_{xA}\sigma_{yA}}\right)\\ \;D=\frac{1}{1-p_{V}^{2}}\left(\frac{\mu_{yV}}{\sigma_{yV}^{2}}-\frac{p_{V\mu_{xV}}}{\sigma_{xV}S_{yV}}\right)+\frac{1}{1-p_{A}^{2}}\left(\frac{\mu_{yA}}{\sigma_{yA}^{2}}-\frac{p_{A\mu_{xA}}}{\sigma_{xA}\sigma_{yA}}\right)\\ \;E=\frac{p_{V}}{\left(1-p_{V}^{2}\right)\sigma_{xV}\sigma_{yV}}+\frac{p_{A}}{\left(1-p_{A}^{2}\right)\sigma_{xA}\sigma_{yA}} \end{array}$$

## Appendix 2

F:(x,y)→(r,θ)

Auditory:

$$\begin{array}{l} F\left(x,y\right)=(\left(-8.79\times 10^{-8}\right)x^{4}+\left(1.27\times 10^{-5}\right)x^{3}-0.0021x^{2}\\ \;+0.0091x+4.51+0.0003y^{3}+0.0053y^{2}\\ \;-0.201y-1.05,g(x)) \end{array}$$

where

$$g\left(x\right)=\left\{\begin{array}{c} \pi /2,x<10\\ 0,x=10\\ -\pi /2,x>10 \end{array}\right\}$$

Visual:

$$\begin{array}{l} F\left(x,y\right)=(\frac{\sqrt{x^{2}+y^{2}}}{11.4}+(-0.001x^{2}-0.0038x+0.0527)\\ \;+\left(-0.0011y^{2}+0.0457y+0.8626\right)\text{​},\\ \;tan^{-1}\left(\frac{-y}{-x}\right)+\left(\frac{\pi x}{270}\right)) \end{array}$$

Bimodal:

$$\begin{array}{l} F\left(x,y\right)=(\frac{\sqrt{x^{2}+y^{2}}}{13.6}+(-0.0014x^{2}+0.0043x+0.7187)\\ \;-\left(1.023\times 10^{-5}\right)y^{4}-\left(2.73\times 10^{-5}\right)y^{3}+0.0036y^{2}\\ \;+0.0364y-0.0463,tan^{-1}\left(\frac{-y}{-x}\right)+\left(\frac{\pi x}{270}\right)) \end{array}$$
